# Uterine C-Kit positive low grade stromal sarcoma

**DOI:** 10.4103/0971-5851.64258

**Published:** 2009

**Authors:** Jovitha Martin, Anita Ramesh, Sarah Kuruvilla, D Lalitha

**Affiliations:** *Departments of Oncology, Sri Ramachandra Medical College, Chennai, India*; 1 *Departments of Pathology, Sri Ramachandra Medical College, Chennai, India*; 2 *Departments of Obstetrics and Gynecology, Sri Ramachandra Medical College, Chennai, India*

**Keywords:** C-Kit, stromal sarcomas, uterine

## Abstract

Uterine C-Kit positive stromal tumors are rare, however, there are a few cases reported in literature. A 58-year-old post menopausal lady presented with bleeding per vaginum. An abdominal examination revealed an enlarged uterus. A computed tomography scan of the abdomen and pelvis showed a large myomatous uterus, with a probable subserosal intramural and intracavitary myoma or cervical myoma in the presence of a solitary large aortocaval node, with multiple bone lesions. The biopsy taken from the uterine mass had revealed, a low-grade uterine sarcoma, which was positive for CD117. This case is presented for its rarity and management dilemma.

## INTRODUCTION

Uterine sarcomas are rare tumors and the common subtypes of uterine sarcomas reported in literature are carcinosarcoma, leiomyosarcoma, rhabdomyosarcoma, adenosarcoma, stromal sarcoma, and undifferentiated sarcoma.[1] Of these subtypes very few cases of Stromal C-Kit positive tumors have been documented. These stromal C-Kit positive uterine sarcomas are characterized by their resistance to chemotherapy and radiation treatment. Surgery is the primary modality of treatment. However, for patients with an unresectable disease, alternate therapeutic options are clearly warranted. In recent times, tyrosine kinase inhibitors such as Imatinib Mesylate, have been investigated for these mesenchymal neoplasms.[2]

## CASE REPORT

A-58-year old multiparous, postmenopausal female, with a regular menstrual history until 2004, started developing bleeding per vaginum and pain in the abdomen, which was insidious on onset and gradual in progression since the last year. She gave a history of hypertension and diabetes mellitus. She was on regular treatment for her comorbidities.

On general examination, her performance status was ECOG[3] (Eastern Cooperative Oncology Group)-3, blood pressure was 150/90 mm Hg, and she had multiple areas of bony tenderness. On Central Nervous System examination she had weakness in both her lower limbs (power of 3/5). Abdominal palpation revealed a soft mobile uterus, 32 weeks in size, which was felt to be rising from the pelvis. On speculum examination, the cervix was not identifiable and a friable growth was seen at the upper end of the vagina. Vaginal examination revealed a 3 × 3 cm indurated growth, with foul smelling discharge, at the upper end of the vagina, with involvement of fornices. On rectal examination, both the parametria were indurated. A differential diagnosis of a disseminated malignancy, with bony metastasis, from a probable endometrial tumor or cervical tumor was suspected at that juncture. An ultrasound of the pelvis showed a thickened, hyperechoic endometrium, 3.4 cm, with uterus measuring 5.7 × 5.6 × 6 cm. A CT of the abdomen and pelvis showed a large myomatous uterus with a probable subserosal intramural and intracavitary myoma or cervical myoma in the presence of a solitary large aortocaval node with multiple bone lesions.

**Figure 1:**
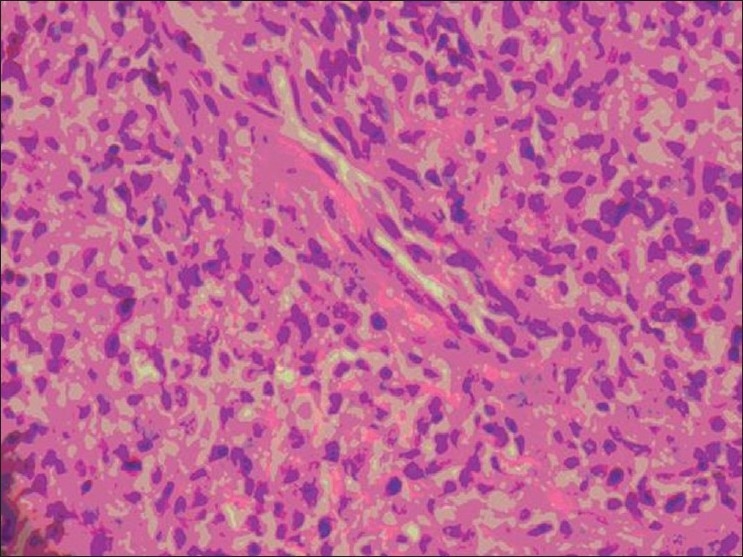
High power microscopy 40×. Histological sections showed a low-grade endometrial uterine sarcoma composed of short, spindleshaped cells, with round-to-oval nuclei, finely clumped chromatin, inconspicuous nucleoli, and eosinophilic cytoplasm and scattered mitotic figures were present

**Figure 2:**
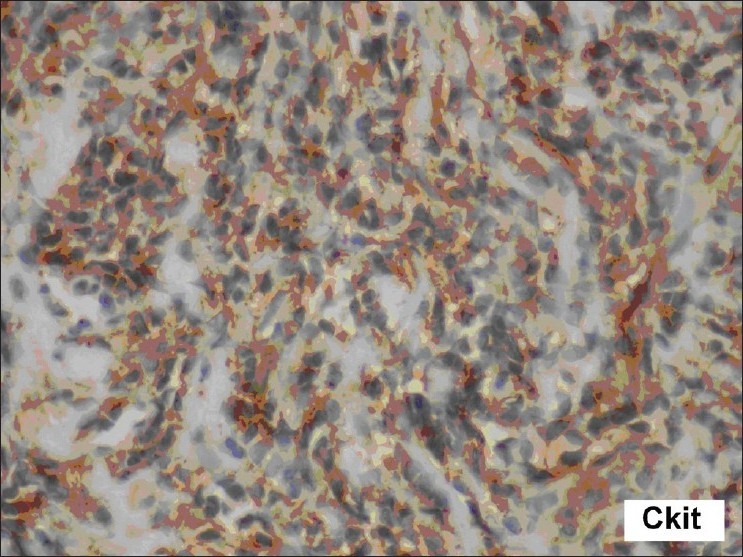
Immunohistochemistry in high power microscopy 40× showed CD117 positivity and CD 10 positivity in the tumor cells

The cervix biopsy [Figure 1] showed a low-grade, endometrial uterine sarcoma, composed of short, spindleshaped cells with round-to-oval nuclei, finely clumped chromatin, inconspicuous nucleoli, and eosinophilic cytoplasm, in the presence of scattered mitotic figures that were present. Immunohistochemistry showed CD117 positivity and CD 10 positivity in the tumor cells [Figure 2]. Urine Bence Jones Protein was negative. Bone marrow biopsy was positive for malignant cells. The Technetium 99 MDP Bone scan revealed multiple bone metastases. She was surgically inoperable; therefore, she was treated with tyrosine kinase inhibitor-Imatinib Mesylate 400 mg, once daily. She had generalized osteoporosis with multiple metastasis, hence, she was given oral Bisphosphonates[4] (Ibandronate 30 mg for 14 days every month) for three months. Palliative local external beam radiotherapy was directed to the secondaries in the Spine from D8 to L12 and the bilateral sacroiliac region, with total dose of 30 Gy in 10 fractions. As she continued to have pain with persistence of vaginal discharge, concurrent palliative chemotherapy with Adriamycin 70 mg and Ifosphamide 5 gm (two cycles) were given, in addition to the oral Imatinib 400 mg once daily. On a subsequent follow-up, the patient had swelling in the left jaw. The repeat bone scan showed increased uptake in the left mandibular region of the jaw, suggestive of metastasis, due to the progressive disease (differentiated from Bisphosphonates-induced osteoradionecrosis, which occurred due to prolonged use of Bisphosphonates or with previous exposure to radiation). After two cycles of Ifosphamide and Adriamycin, the chemotherapy regimen was changed to Paclitaxol 260 mg and Gemcitabine 1000 mg as the disease was progressive. The dose of Imatinib Mesylate was not escalated as the patient did not respond to Imatinib, and it was used either as a single agent or used with combination chemotherapy. Due to financial constraints the genetic tests for exon 9 or other mutations were not done and the next choice of Sunitinib was opted out. Although Imatinib Mesyate and Ibandronate were continued, she had progressive disease within a short duration of time and succumbed to her illness.

## DISCUSSION

Uterine leiomyosarcomas and stromal tumors are rare tumors.[5] Their symptomatology is non-specific and they are characterized by histopathological diversity.[1] A literature review showed that around 50 cases of Uterine C-Kit positive stromal sarcomas had been documented.[5–8]

C-Kit is expressed in most gastrointestinal stromal tumors, and they usually show c-kit aberrations (most frequently deletions or deletions coexisting with a single or multiple point mutations).[6] In recent times, several studies regarding KIT expression in gynecological tumors have been reported; however, their outcomes were not consistent. According to the initial observations of C-KIT expression, correlation with a bad prognosis, and the successful therapeutic possibility of STI571 in gastrointestinal stromal tumors, along with the data has encouraged us to study C-KIT expression in these tumors.[5] The proto-oncogene C-Kit encodes a 145-kDa transmembrane tyrosine kinase receptor. Interaction with its ligand stem cell factor is essential in the development of hematopoietic stem cells, mast cells, gametocytes, melanocytes, and interstitial cells of Cajal.[9] C-kit expression has been identified in a number of different neoplasms that include mastocytosis / mast cell leukemia, acute myeloblastic leukemia, seminoma / dysgerminoma, and gastrointestinal stromal tumors.

Many uterine sarcomas express one or more of the kinases targeted by Imatinib Mesylate such as C-Kit, abl and platelet-derived growth factor receptor-beta (PDGFR-beta).[7] Of late, the use of tyrosine kinase inhibitors, such as STI-571, has resulted in the successful treatment of bcr-abl-positive leukemias and gastrointestinal stromal tumors.[8] Due to its rarity, the proper treatment consensus has not yet evolved and hence, early diagnosis is essential, as the patients’ survival is correlated to the tumor stage.[1] These tumors are characterized by their resistance to chemotherapy and radiation treatment. Surgery is the primary modality of treatment, but for patients with unresectable disease, alternate therapeutic options are clearly necessary. The use of tyrosine kinase inhibitors (TKIs) has resulted in the successful treatment of C-KIT-positive neoplasms.[10] Consequently, c-kit expression may have significant clinical implications for this tumor.

These low grade C-Kit uterine sarcomas are clinically aggressive and their natural history is unknown. In this case, Imatinib Mesylate was used, as the patient was diagnosed. As there was not much relief from the local symptoms; such as, bleeding and discharge per vaginum, Imatinib Mesylate in combination with a compromised dose of palliative chemotherapy were given in view of the poor performance status of the patient. However, there was rapid disease progression (in a short duration of time less than six months), which was resistant to chemotherapy. Hence, further studies based on the natural history of the tumor and the investigation of Tyrosine Kinase Inhibitors (Imatinib Mesylate), as a therapy for uterine c-kit positive stromal sarcomas is warranted. The evolution of an appropriate consensus for the treatment of these tumors is awaited.[7]
